# Two-way learning with one-way supervision for gene expression data

**DOI:** 10.1186/s12859-017-1564-5

**Published:** 2017-03-04

**Authors:** Monica H. T. Wong, David M. Mutch, Paul D. McNicholas

**Affiliations:** 1 Department of Mathematics and Statistics, McMaster University, Hamilton, L8S 4L8 ON Canada; 2 Department of Human Health and Nutritional Sciences, University of Guelph, Guelph, N1G 2W1 ON Canada

**Keywords:** Biclustering, Biomarker discovery, Finite mixture models, Microarray gene expression, Surrogate tissue

## Abstract

**Background:**

A family of parsimonious Gaussian mixture models for the biclustering of gene expression data is introduced. Biclustering is accommodated by adopting a mixture of factor analyzers model with a binary, row-stochastic factor loadings matrix. This particular form of factor loadings matrix results in a block-diagonal covariance matrix, which is a useful property in gene expression analyses, specifically in biomarker discovery scenarios where blood can potentially act as a surrogate tissue for other less accessible tissues. Prior knowledge of the factor loadings matrix is useful in this application and is reflected in the one-way supervised nature of the algorithm. Additionally, the factor loadings matrix can be assumed to be constant across all components because of the relationship desired between the various types of tissue samples. Parameter estimates are obtained through a variant of the expectation-maximization algorithm and the best-fitting model is selected using the Bayesian information criterion. The family of models is demonstrated using simulated data and two real microarray data sets. The first real data set is from a rat study that investigated the influence of diabetes on gene expression in different tissues. The second real data set is from a human transcriptomics study that focused on blood and immune tissues. The microarray data sets illustrate the biclustering family’s performance in biomarker discovery involving peripheral blood as surrogate biopsy material.

**Results:**

The simulation studies indicate that the algorithm identifies the correct biclusters, most optimally when the number of observation clusters is known. Moreover, the biclustering algorithm identified biclusters comprised of biologically meaningful data related to insulin resistance and immune function in the rat and human real data sets, respectively.

**Conclusions:**

Initial results using real data show that this biclustering technique provides a novel approach for biomarker discovery by enabling blood to be used as a surrogate for hard-to-obtain tissues.

**Electronic supplementary material:**

The online version of this article (doi:10.1186/s12859-017-1564-5) contains supplementary material, which is available to authorized users.

## Background

With the introduction of personalized medicine, the discovery of novel biomarkers via “omics” research plays a critical role in its advancement [1]. A biomarker is defined as “a characteristic that is objectively measured and evaluated as an indicator of normal biological processes, pathogenic processes, or pharmacologic responses to a therapeutic intervention” [2]. The behaviour of a biomarker is expected to vary among individuals, thereby allowing treatment to be “personalized” depending on that individual’s (predicted) response. The ideal diagnostic tool is minimally invasive, leading researchers to investigate the use of peripheral blood cells as surrogate biopsy material, since blood is more easily accessible. The assumption is that the molecular profile of peripheral blood reflects a global overview of the physiological events occurring in different tissues throughout the body [3].

When gene expression microarrays are used for biomarker discovery, the subset of identified genes acts as the set of biomarkers [4, 5]. Returning to the idea of peripheral blood as surrogate material, a gene that exhibits correlated expression profiles in blood and a given tissue (but not other tissues) may be a biomarker of interest. In this scenario, the genes act as the observations and the blood and tissues (the samples) act as the variables. A data point in the microarray data set is thus an intensity value corresponding to a specific gene in a sample. One popular way of identifying these subsets of correlated genes across the blood and the given tissue is via clustering techniques [6].

One-way clustering methods can be restrictive in certain applications. It is not always the case that the groups of patterns found in the observations are homogeneous across all the variables; rather, it could be the case that only a subset of the variables possesses these groupings. With gene expression data, if the samples are the variables and the researcher hypothesizes that there exists homogeneous groups of samples, this would be useful information for the algorithm to have. A popular example is the discovery of leukaemia tumour subtypes based on gene expression [7]. Consequently, biclustering techniques have been developed to address this recurring issue. Biclustering, first explored by Hartigan [8], clusters both rows and columns simultaneously and results in biclusters.

Biclustering is a useful technique when the researcher suspects biclusters of variables and observations in the data, but does not understand what properties of the variables define the biclusters. For instance, in the leukaemia tumour subtype analysis, researchers initially would not have known the classes of each tumour sample (see [7] for a discussion). Here, biclustering could help to reveal these subtypes more efficiently, as done by Kluger et al. [9] for example. However, researchers may desire that the observations within biclusters satisfy a particular relationship among the variables; the biclustering method would then be one-way supervised. This technique is particularly relevant for the blood biomarker discovery application mentioned earlier. One-way supervision is effective because the researcher specifically requires a prominent relationship between the samples of blood and the samples of the tissue of interest with respect to the expression profile of a subset of genes. Additionally, the researcher explicitly requires that the expression profiles of that same subset of genes to have no relationship between the previously mentioned samples and the rest of the samples in the data set. In this way, the resulting biclusters would contain a subset of genes that is strictly correlated within blood and the tissue of interest only.

### Model-based clustering

Cluster analysis identifies homogeneous groups that are relevant within a population. It is an unsupervised technique because it does not utilize existing labels to find the best homogeneous groups among a set of observations, which reflects common real-life scenarios because observations are not usually accompanied by hints about their true groupings with respect to the variables. Some popular clustering techniques include methods such as hierarchical clustering [10], *k*-means clustering [11], and model-based clustering (see [12] for an in-depth discussion).

In model-based clustering, group membership is estimated using a parametric finite mixture model, which can be denoted

1 $$ f(\mathbf{x}\mid\boldsymbol{\vartheta}) = \sum\limits_{g=1}^{G} \pi_{g}f_{x}\left(\mathbf{x}\mid\boldsymbol{\theta}_{g}\right),  $$

where *π*_*g*_∈(0,1], such that $\sum _{g=1}^{G} \pi _{g} = 1$, is the mixing proportion for component *g*, *f*_*x*_(**x**∣***θ***_*g*_) is the density of a multivariate random variable **X** with parameters ***θ***_*g*_, and ***𝜗***=(*π*_1_,…,*π*_*G*_,***θ***_1_,…,***θ***_*G*_). Frequently, the finite Gaussian mixture model is used because of its mathematical tractability. This density is given by

2 $$ f(\mathbf{x}\mid\boldsymbol{\vartheta}) = \sum\limits_{g=1}^{G} \pi_{g}\phi\left(\mathbf{x}\mid\boldsymbol{\mu}_{g}, \boldsymbol{\Sigma}_{g}\right),  $$

where *ϕ*(**x**∣***μ***_*g*_,***Σ***_*g*_) is the density of a multivariate Gaussian random variable **X** with mean ***μ***_*g*_ and covariance matrix ***Σ***_*g*_. An overview of model-based clustering is given by McNicholas [13].

### Parsimonious Gaussian mixture models

The factor analysis model [14], assumes that a *p*-dimensional random vector **X**_*i*_ can be modelled using a *q*-dimensional vector of latent factors **U**_*i*_, where *q*<*p*. Factor analysis allows for a decrease in the number of parameters, which is useful in high-dimensional data cases. The model can be written as

3 $$ \mathbf{X}_{i} = \boldsymbol{\mu} + \boldsymbol{\Lambda U}_{i} + \boldsymbol{\epsilon}_{i},  $$

where ***Λ*** is a *p*×*q* matrix of factor loadings, the latent factors **U**_*i*_∼*N*(**0**,**I**_*q*_) are independent, and the errors ***ε***_*i*_∼*N*(**0**,***Ψ***) are independently distributed and independent of the **U**_*i*_, where ***Ψ*** is a diagonal noise matrix with dimensions *p*×*p*. Thus, **X**_*i*_∼*N*(***μ***,***Λ*** ***Λ*** ^′^+***Ψ***). In the mixture of factor analyzers (MFA) model, different factor analysis models are allowed in different regions of the feature subspace, using the density of a Gaussian mixture model with covariance structure ***Σ***_*g*_=***Λ***_*g*_ ***Λ*** *g*′+***Ψ*** [15] or ***Σ***_*g*_=***Λ***_*g*_ ***Λ*** *g*′+***Ψ***_*g*_ [16]. The mixture of probabilistic principal components analysis (PPCA) model [17] is a special case of the MFA model from [16] in that it adds the assumption that the noise matrix is isotropic so that ***Ψ***_*g*_=*ψ*_*g*_ **I**_*p*_. The parsimonious Gaussian mixture model (PGMM) family [18] allows combinations of the constraints: ***Λ***_*g*_=***Λ***, ***Ψ***_*g*_=***Ψ***, and ***Ψ***_*g*_=*ψ*_*g*_ **I**_*p*_ within the MFA model, resulting in a family of eight models.

### Model-based biclustering

A recent review of biclustering on expression data by Pontes et al. [19] classifies the methods using various taxonomies. One taxonomy is based on bicluster structure, specifically whether or not the genes and/or samples must be assigned to a bicluster (exhaustivity) and whether or not the genes and/or samples can be assigned to multiple biclusters (exclusivity). When considering blood biomarker discovery, an implicit property of the biomarker is that its expression profile is highly correlated between the blood and tissue of interest, and distinct from the rest of the tissues; indicating a unique biomarker for that tissue. Thus, the researcher would be interested in samples that are assigned to one bicluster only, in other words, non-overlapping column-exclusive biclusters. Examples of existing biclustering methods that adopt this property are plaid models developed by Lazzeroni and Owen [20], biclustering via Gibbs sampling developed by Sheng et al. [21], and Bayesian biclustering developed by Gu and Liu [22]. These are also examples of non metric-based probabilistic biclustering methods, based on another taxonomy provided in the review. The reader is referred to the review paper by Pontes et al. [19] for a structured and detailed discussion on the available biclustering methods.

Under the probabilistic framework, Martella et al. [23] propose a modified MFA technique for high-dimensional data for simultaneously clustering observations and variables. Variable cluster membership is represented by a binary row-stochastic matrix, which can be estimated via

$${}\begin{aligned} \hat{\boldsymbol{\Lambda}}_{g} = \left\{\lambda_{gjl}\right\} = \left\{\begin{array}{ll} 1 & \text{if}~Q(\cdot,\lambda_{gjl}=1) = \text{max}_{h}Q(\cdot,\lambda_{gjh}=1),\\ 0 & \text{otherwise}, \end{array}\right. \end{aligned} $$

where *j*=1,…,*p*, *h,l*=1,…,*q*, *g*=1,…,*G*, and *Q* is the expected complete-data log-likelihood. We have **X**_*i*_=***μ***_*g*_+***Λ***_*g*_ **U**_*ig*_+***ε***_*ig*_ with probability *π*_*g*_. In this case, **U**_*ig*_∼*N*(**0**,**I**_*q*_) and **X**_*i*_∼*N*(***μ***_*g*_,***Λ***_*g*_ ***Λ*** *g*′+***Ψ***_*g*_). This particular form of factor loadings matrix results in a block-diagonal covariance matrix which is especially suitable in the biclustering framework because it models the grouped nature of the variables. Additionally, it results in the non-overlapping biclusters that are useful in blood biomarker discovery. Constraining or not constraining the covariance parameters across clusters leads to a family of four models. This family will be referred to as MFABC from this point forward. The remainder of this paper describes a one-way supervised biclustering technique and its application to simulated and real data.

## Methods

### Covariance structure

To accommodate biclustering we set the factor loadings matrix to be binary row-stochastic. To allow for supervision along the variable dimension, we provide the structure of this matrix to the algorithm. In our gene expression analysis case, the variables are the samples, thus we are setting a relationship between the samples in the data set and providing it to the algorithm during initialization and take it as constant. Constraints can be imposed or not on ***Λ***_*g*_, ***Ψ***_*g*_, and ***Ψ***_*g*_=*ψ*_*g*_ **I**_*p*_ to create a family of eight one-way-supervised Gaussian mixture models for biclustering (Table 1), which will be referred to as OSGaBi (**o**ne-way **s**upervised **Ga**ussian **bi**clustering) hereafter.

**Table 1:** Properties of the OSGaBi family

Model nomenclature			Covariance structure (***Σ***_*g*_)	Covariance parameters
***Λ***_*g*_=***Λ***	***Ψ***_*g*_=***Ψ***	***Ψ***_*g*_=*ψ*_*g*_ **I**_*p*_		
C	C	C	***Λ*** ***Λ*** ^′^+*ψ* **I**_*p*_	1
C	C	U	***Λ*** ***Λ*** ^′^+***Ψ***	*p*
C	U	C	***Λ*** ***Λ*** ^′^+*ψ*_*g*_ **I**_*p*_	*G*
C	U	U	***Λ*** ***Λ*** ^′^+***Ψ***_*g*_	*Gp*
U	C	C	***Λ***_*g*_ ***Λ*** *g*′+*ψ* **I**_*p*_	1
U	C	U	***Λ***_*g*_ ***Λ*** *g*′+***Ψ***	*p*
U	U	C	***Λ***_*g*_ ***Λ*** *g*′+*ψ*_*g*_ **I**_*p*_	*G*
U	U	U	***Λ***_*g*_ ***Λ*** *g*′+***Ψ***_*g*_	*Gp*

The nomenclature, covariance structure, and number of covariance parameters for each member of the OSGaBi family. C, constrained; U, unconstrained

### Parameter estimation

This section provides the mathematical details required to compute the parameter estimates for the eight members of the OSGaBi family, with a focus on the CUU model because it is the most appropriate model for the application presented previously and later in the Application section. The expectation-maximization (EM) algorithm [24] is an iterative procedure for computing the maximum likelihood estimates (MLE) when data are incomplete or treated as such. The EM algorithm is based on the complete-data, which consist of both observed and missing data. The algorithm begins with the expectation step (E-step), where the expected value of the complete-data log-likelihood (*Q*) is computed conditional on the current parameter estimates. In the maximization step (M-step), *Q* is maximized with respect to the model parameters. These two steps are repeated until convergence.

The alternating expectation-conditional maximization (AECM) algorithm [25] incorporates a series of conditional maximization (CM) steps instead of a single M-step and also allows for different specification of the complete-data at each stage. This algorithm is used for parameter estimation for the MFA model, the PGMM family, and the MFABC family. It will also be used for the OSGaBi family.

For convenience, the following notation is adopted. We denote the observed data as **x** and the unobserved latent parameters as **U**_*i*_=(**U**_*i*1_,…,**U**_*iG*_). We denote the missing group memberships as **z**_*i*_, where

$$z_{ig} = \left\{\begin{array}{ll} 1 & \text{if observation}\, i\, \text{belongs to component}\, {g},\\ 0 & \text{otherwise}, \end{array}\right. $$

for *i*=1,…,*n*, *g*=1,…,*G*.

In the first cycle of the AECM algorithm, (**x**_*i*_,**z**_*i*_) are the complete-data, where *i*=1,…,*n*. During the CM-step, *π*_*g*_ and ***μ***_*g*_ are updated. During the E-step, the *z*_*ig*_ are replaced by their expected values

$${}\begin{aligned} \mathbb{E}[\!Z_{ig}\mid\hat{\pi}_{g},\hat{\boldsymbol{\mu}}_{g},\hat{\boldsymbol{\Lambda}}_{g},\hat{\boldsymbol{\Psi}}_{g}] \! = \! \frac{\hat{\pi}_{g}\phi(\mathbf{x}_{i}\mid\hat{\boldsymbol{\mu}}_{g},\hat{\boldsymbol{\Lambda}}_{g},\hat{\boldsymbol{\Psi}}_{g})}{\sum_{h=1}^{G}\hat{\pi}_{h}\phi(\mathbf{x}_{i}\mid\hat{\boldsymbol{\mu}}_{h},\hat{\boldsymbol{\Lambda}}_{h},\hat{\mathbf{\Psi}}_{h})} \! = : \hat{z}_{ig} \end{aligned} $$

leading to the calculation of the expected value of the complete-data log-likelihood, *Q*_1_. In the CM-step, *Q*_1_ is maximized to give

$$\hat{\pi}_{g} = \frac{n_{g}}{n}$$

and

$$\hat{\boldsymbol{\mu}}_{g} = \frac{\sum_{i=1}^{n} \hat{z}_{ig}\mathbf{x}_{i}}{n_{g}}, $$

where $n_{g} = \sum _{i=1}^{n} \hat {z}_{ig}$.

In the second cycle of the AECM algorithm, (**x**_*i*_,**z**_*i*_,**U**_*i*_) are the complete-data. During this CM-step, ***Ψ***_*g*_ is updated. During this E-step, *z*_*ig*_ are replaced by $\hat {z}_{ig}$ and **U**_*ig*_ and **U**_*ig*_ **U** *ig*′ are replaced by

4 $$ {}\begin{aligned} \mathbb{E}\left[\mathbf{U}_{ig}\mid\mathbf{x}_{i},\boldsymbol{\mu}_{g},\boldsymbol{\Lambda},\boldsymbol{\Psi}_{g}\right] & = \boldsymbol{\beta}_{g}\sum\limits_{i=1}^{n} \hat{z}_{ig}(\mathbf{x}_{i}-\boldsymbol{\mu}_{g}),\\ \mathbb{E}\left[\mathbf{U}_{ig}\mathbf{U}'_{ig}\mid\mathbf{x}_{i},\boldsymbol{\mu}_{g},\boldsymbol{\Lambda},\boldsymbol{\Psi}_{g}\right] & = \mathbf{I}_{q}-\boldsymbol{\beta}_{g}\boldsymbol{\Lambda}\\ & ~~ +\boldsymbol{\beta}_{g}\!\sum\limits_{i=1}^{n}\hat{z}_{ig}(\mathbf{x}_{i}\! -\!\boldsymbol{\mu}_{g})(\mathbf{x}_{i}\! -\!\boldsymbol{\mu}_{g})'\!\boldsymbol{\beta}'_{g} \end{aligned}  $$

respectively, where ***β***_*g*_=***Λ*** ^′^(***Λ*** ***Λ*** ^′^+***Ψ***_*g*_)^−1^ for model CUU, to allow for the calculation of *Q*_2_. In the CM-step, the maximization of *Q*_2_ is specific for each model. Considering the CUU model,

5 $$ \begin{aligned} Q_{2}(\boldsymbol{\Lambda},\boldsymbol{\Psi}_{g}) &= C + \sum\limits_{g=1}^{G} \frac{n_{g}}{2}\left[\log|\boldsymbol{\Psi}_{g}^{-1}|-\text{tr}\left\{\boldsymbol{\Psi}_{g}^{-1}\mathbf{S}_{g}\right\}\right.\\ & \quad +2\text{tr}\left\{\boldsymbol{\Psi}_{g}^{-1}\boldsymbol{\Lambda}\boldsymbol{\beta}_{g}\mathbf{S}_{g}\right\}\\ &\quad \left.-\text{tr}\left\{\boldsymbol{\Psi}_{g}^{-1}\boldsymbol{\Lambda}\boldsymbol{\Theta}_{g}\boldsymbol{\Lambda}'\right\}\right], \end{aligned}  $$

where *C* is a constant with respect to the unknown parameters, $\mathbf {S}_{g}=\frac {1}{n_{g}}\sum _{i=1}^{n} \hat {z}_{ig}(\mathbf {x}_{i}-\boldsymbol {\mu }_{g})(\mathbf {x}_{i}-\boldsymbol {\mu }_{g})'$, and ***Θ***_*g*_=**I**_*q*_−***β***_*g*_ ***Λ***+***β***_*g*_ **S**_*g*_ ***β*** *g*′.

The following score function is obtained when differentiating *Q*_2_ with respect to ***Ψ***_*g*_:

6 $$ \begin{aligned} S(\boldsymbol{\Lambda},\boldsymbol{\Psi}_{g}) & = \frac{\delta Q}{\delta\boldsymbol{\Psi}_{g}^{-1}}\\ &=\sum\limits_{g=1}^{G}\frac{n_{g}}{2}\Big[\boldsymbol{\Psi}_{g}-\mathbf{S}_{g}+2\boldsymbol{\Lambda}\boldsymbol{\beta}_{g} \mathbf{S}_{g}-\boldsymbol{\Lambda}\boldsymbol{\Theta}_{g}\boldsymbol{\Lambda}'\Big]. \end{aligned}  $$

Now, setting $S(\boldsymbol {\Lambda },\hat {\boldsymbol {\Psi }}_{g}) = 0$ and solving gives the estimate

$$\begin{array}{*{20}l} \hat{\boldsymbol{\Psi}}_{g} = \text{diag}\left\{\mathbf{S}_{g}-2\boldsymbol{\Lambda}\boldsymbol{\beta}_{g}\mathbf{S}_{g}+\boldsymbol{\Lambda}\boldsymbol{\Theta}_{g}\boldsymbol{\Lambda}'\right\}. \end{array} $$

The parameter estimates for the remaining seven models are derived similarly and are provided in the Additional file 1 titled OSGaBi_MWong_appendix.pdf.

When running the AECM algorithm, utilizing the Woodbury identity [26] avoids inverting any non-diagonal *p*×*p* matrices that may be singular for *p*≫*n*. Suppose an *n*×*n* matrix **A**, an *n*×*q* matrix **H**, a *q*×*q* matrix **C**, and a *q*×*n* matrix **V**. The Woodbury identity states that

7 $$ (\mathbf{A}+\mathbf{HCV})^{-1} = \mathbf{A}^{-1}-\mathbf{A}^{-1}\mathbf{H}(\mathbf{C}^{-1}+\mathbf{VA}^{-1}\mathbf{H})^{-1}\mathbf{VA}^{-1}.  $$

Specifically for the AECM algorithm, setting **H**=***Λ***, **V**=***Λ*** ^′^, **A**=***Ψ***, and **C**=**I**_*q*_ results in

8 $$ (\boldsymbol{\Psi}+\boldsymbol{\Lambda}\boldsymbol{\Lambda}')^{-1} = \boldsymbol{\Psi}^{-1}-\boldsymbol{\Psi}^{-1}\boldsymbol{\Lambda}(\mathbf{I}_{q}+\boldsymbol{\Lambda}'\boldsymbol{\Psi}^{-1}\boldsymbol{\Lambda})^{-1}\boldsymbol{\Lambda}'\boldsymbol{\Psi}^{-1}.  $$

Now, instead of inverting the *p*×*p* covariance matrix on the left side of Eq. 8, only the diagonal and *q*×*q* matrices on the right side need to be inverted. With gene expression data where *q*≪*p*, this identity provides a major computational advantage. Another useful identity is for calculating the determinant of the covariance matrix in the AECM algorithm:

$$\begin{array}{*{20}l} |\boldsymbol{\Psi}+\boldsymbol{\Lambda}\boldsymbol{\Lambda}'| = \frac{|\boldsymbol{\Psi}|}{|\mathbf{I}_{q}-\boldsymbol{\Lambda}'(\boldsymbol{\Lambda}\boldsymbol{\Lambda}'+\boldsymbol{\Psi})^{-1}\boldsymbol{\Lambda}|}. \end{array} $$

### Component membership

The predicted biclustering for each member of the OSGaBi family is given by the maximum *a posteriori* (MAP) classification for the observations and the classifications originally provided for the variables. That is, the posterior predicted component membership of observation (i.e., gene) *i* is the value of *g* for which $\hat {z}_{ig}$ is greatest. In the biological sense, this will identify which gene belongs to which subset, implying that the genes in each subset are related in some way. Component membership of variable (i.e., sample) *j* is already provided as ***Λ***_*g*_ at the beginning of the algorithm, specifically

$$\boldsymbol{\Lambda}_{g} = \left\{\lambda_{gjl}\right\} = \left\{\begin{array}{ll} 1 & \text{if variable}\, {j}\, \text{belongs to cluster}\, {l},\\ 0 & \text{otherwise}, \end{array}\right. $$

for *j*=1,…,*p*, *l*=1,…,*q*, and *g*=1,…,*G*. In the biological view, we know a priori that a certain set of samples are/should be related to each other, which is uncorrelated to another set of samples. A concrete example of how component membership is applied in microarray gene expression analysis is presented in the Application section.

### Convergence and model selection

Convergence of the AECM algorithm is determined using the Aitken’s acceleration [27] to estimate the asymptotic maximum of the log-likelihood at each iteration of the AECM algorithm for a specific number of components and a specific number of factors, as described in [28]. The Aitken’s acceleration at iteration *t* is

$$a^{(t)} = \frac{l^{(t+1)}-l^{(t)}}{l^{(t)}-l^{(t-1)}}, $$

where *l* corresponds to the respective log-likelihood. The asymptotic estimate of the log-likelihood at iteration *t*+1 is

$$l^{(t+1)}_{\infty} = l^{(t)}+\frac{1}{1-a^{(t)}}\left(l^{(t+1)}-l^{(t)}\right) $$

[29]. The stopping criterion $l^{(t+1)}_{\infty }-l^{(t)}<\epsilon $ [30], where *ε*=0.1, is used and provided that the difference is positive [13]. The Bayesian information criterion (BIC) [31] is used to choose the best member of the proposed OSGaBi family with respect to the model and number of components, *G*.

## Results

### Simulation studies

Simulation studies were carried out to validate the proposed biclustering algorithm. The adjusted Rand index (ARI) [32] was used to evaluate the performance of the algorithm in recovering biclusters from the simulated data. Specifically, **z**_*i*_ was compared with $\hat {\mathbf {z}}_{i}$ after convergence was attained. Model selection was done via the BIC as previously described, although it can be noted that the integrated completed likelihood (ICL) [33] and Akaike information criterion (AIC) [34] were used as comparison and produced the same outcomes. The parameters and resulting data sets for the following simulation studies are found in the (Additional file 1: Supplementary files).

**Fig. 1:**
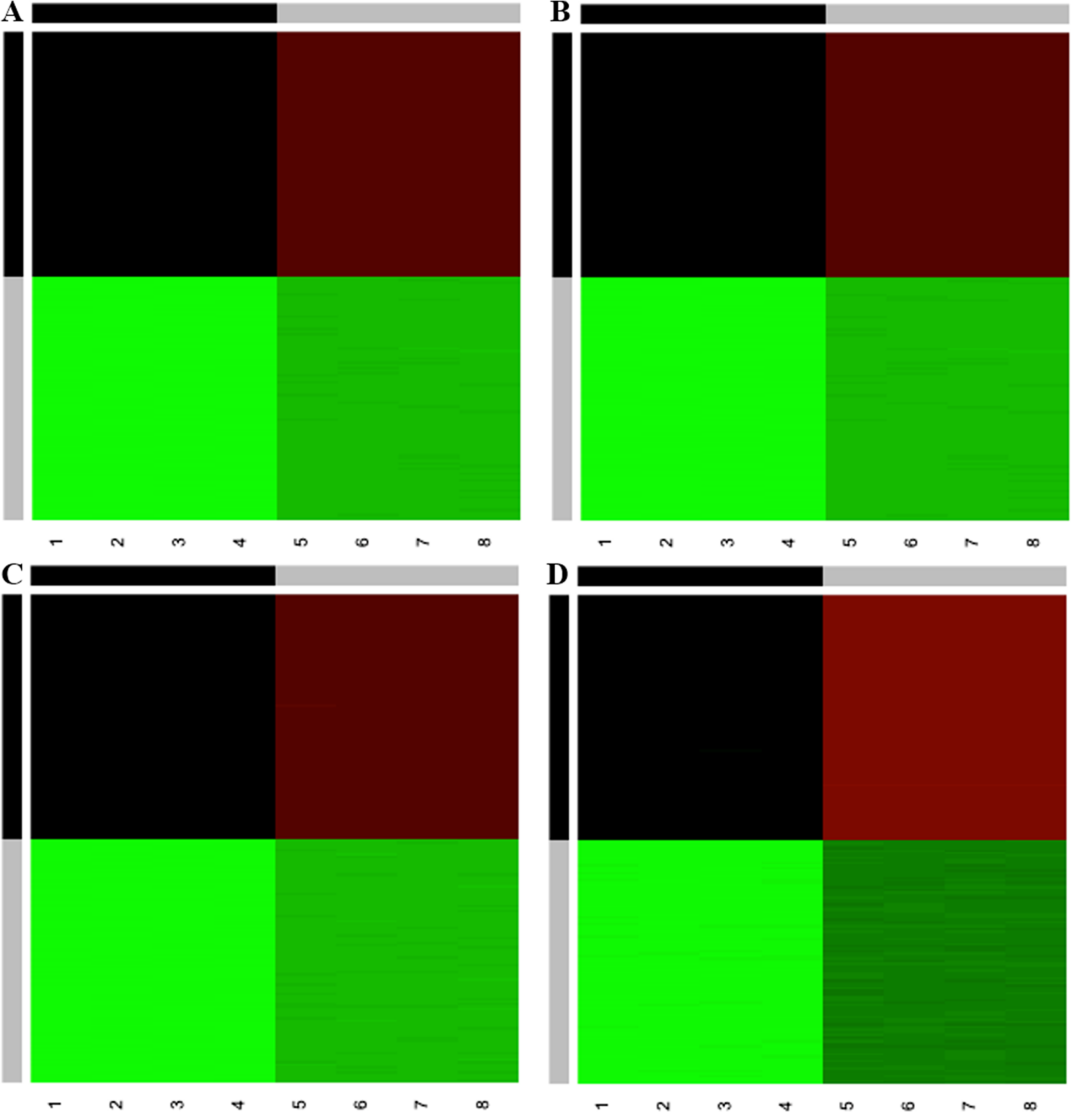
Heatmaps of simulated data for CUC model. Examples of heatmaps of the four types of simulated data used for the CUC model: low variance and good cluster separation (**a**), medium variance and good cluster separation (**b**), high variance and good cluster separation (**c**), and high variance with relatively close clusters (**d**)

Simulated data were generated with *G*= 2, 3, and 4 clusters for observations and *q*=2 clusters for variables. This resulted in 4, 6, and 8 biclusters, respectively. Four cases were examined: low, medium, and high variance coupled with good cluster separation, and high variance coupled with relatively close clusters. For each case, 100 data sets were generated, where each set had *p*=8 variables and *n*=200,300,400 observations (for *G*=2,3,4, respectively) and was randomly generated from the same Gaussian distribution. Examples of heatmaps for each of the CUC cases visibly indicate that there are distinct biclusters in the simulated data (Fig. 1). To reflect the one-way supervised nature of the algorithm, the true ***Λ*** was provided. Twenty random starts were used for each run of the algorithm. Table 2 presents the results from these four simulation studies for the CUU and CUC models. It lists the average number of components selected, the most frequently chosen model, and the average ARI when fitting *G*=2,…,10 observation clusters. Because the algorithm was sometimes overfitting for the number of components based on the model it chose, another analysis was included to show the average ARI when the number of clusters was known (i.e., *G*= 2, 3, 4, depending on the case). These results are shown in the last column. The CUU and CUC models are focused on because they are the most probable cases in real-life scenarios, and additionally, they are the models most frequently selected when the number of clusters was known (results not shown).

**Table 2:** Simulation study results for model CUU

	*G*=2,…,10			*G*=*g*_known_
Case	Average *G*	Most chosen model	Average ARI	Average ARI
CUC, *g*_known_=2				
Low var, good cluster sep	2.5 (0.8)	CUU	0.955 (0.083)	1.0 (0.0)
Mid var, good cluster sep	2.4 (0.8)	CUU	0.955 (0.094)	1.0 (0.0)
High var, good cluster sep	4.0 (1.0)	CUC	0.708 (0.106)	1.0 (0.0)
High var, close clusters	6.4 (1.2)	CUC	0.502 (0.135)	1.0 (0.0)
CUU, *g*_known_=2				
Low var, good cluster sep	2.4 (0.7)	CUU	0.969 (0.072)	1.0 (0.0)
Mid var, good cluster sep	2.5 (0.8)	CUU	0.964 (0.071)	1.0 (0.0)
High var, good cluster sep	4.0 (1.0)	CUC	0.705 (0.103)	1.0 (0.0)
High var, close clusters	6.4 (1.3)	CUC	0.485 (0.138)	1.0 (0.0)
CUC, *g*_known_=3				
Low var, good cluster sep	3.5 (0.7)	CUU	0.981 (0.039)	1.0 (0.0)
Mid var, good cluster sep	3.4 (0.7)	CUU	0.984 (0.033)	1.0 (0.0)
High var, good cluster sep	5.1 (1.0)	CUC	0.864 (0.081)	1.0 (0.0)
High var, close clusters	8.8 (1.1)	CCC	0.601 (0.066)	1.0 (0.0)
CUU, *g*_known_=3				
Low var, good cluster sep	3.5 (0.6)	CUU	0.984 (0.028)	1.0 (0.0)
Mid var, good cluster sep	3.4 (0.7)	CUU	0.975 (0.050)	1.0 (0.0)
High var, good cluster sep	5.0 (1.1)	CUC	0.866 (0.079)	1.0 (0.0)
High var, close clusters	8.8 (1.0)	CUC	0.590 (0.070)	1.0 (0.0)
CUC, *g*_known_=4				
Low var, good cluster sep	4.4 (0.7)	CUU	0.989 (0.254)	1.0 (0.0)
Mid var, good cluster sep	4.3 (0.5)	CUU	0.992 (0.020)	1.0 (0.0)
High var, good cluster sep	6.2 (1.0)	CUC	0.887 (0.048)	1.0 (0.0)
High var, close clusters	9.7 (0.5)	CUC	0.658 (0.045)	1.0 (0.0)
CUU, *g*_known_=4				
Low var, good cluster sep	4.4 (0.9)	CUU	0.989 (0.031)	1.0 (0.0)
Mid var, good cluster sep	4.4 (0.7)	CUU	0.989 (0.024)	1.0 (0.0)
High var, good cluster sep	4.6 (0.8)	CUU	0.970 (0.048)	1.0 (0.0)
High var, close clusters	9.8 (0.5)	CUC	0.653 (0.046)	1.0 (0.0)

Average ARI, most frequently chosen model, and the number of observation clusters selected for the CUU and CUC models using simulated data with low, medium, and high variance (var) with good cluster separation (sep), and high variance with relatively close clusters when fitting *G*=2,…,10 observation clusters using 100 data sets and 20 random starts. The last column presents the ARI when fixing *G*=*g*_known_, where *g*_known_ represents the number of observation clusters the data was generated from. Values in brackets represent the respective standard deviation

For completeness, simulation studies were conducted on the remaining six OSGaBi models using simulated data with medium variance and good cluster separation and with the same properties as that used for the CUU and CUC models. As the true ***Λ***_*g*_ or ***Λ*** was provided, it implied that for models with unconstrained ***Λ*** (i.e., models UUU, UUC, UCU, and UCC), *G* was known because the ***Λ*** for each component would have been provided. Table 3 presents the average ARI, most frequently chosen model, and average number of clusters selected from this simulation study for each of the remaining six models when fitting *G*=2,…,10 clusters for observations. Because the algorithm was once again sometimes overfitting for the number of components based on the model selected, this final analysis was included to show the algorithm’s performance when the number of clusters was fixed to *G*=*g*_known_, where *g*_known_ represents the number of observation clusters the data was generated from. The last column of the table presents the corresponding average ARI when fixing *G* for the CCU and CCC models.

**Table 3:** Simulation study results for the other six models

	*G*=2,…,10			*G*=*g*_known_
Model	Average *G*	Most chosen model	Average ARI	Average ARI
*g*_known_ = 2				
UUU	2	UUC	1.0 (0.0)	NA
UUC	2	UUC	1.0 (0.003)	NA
UCU	2	UCC	0.960 (0.028)	NA
UCC	2	UCC	0.961 (0.025)	NA
CCU	2.3 (0.7)	CCU	0.971 (0.098)	1.0 (0.0)
CCC	2.4 (1.2)	CCU	0.959 (0.113)	1.0 (0.0)
*g*_known_ = 3				
UUU	3	UUC	1.0 (0.0)	NA
UUC	3	UUC	1.0 (0.0)	NA
UCU	3	UCC	1.0 (0.0)	NA
UCC	3	UCC	1.0 (0.0)	NA
CCU	4.0 (1.3)	CCU	0.936 (0.095)	1.0 (0.0)
CCC	4.3 (1.4)	CCU	0.915 (0.107)	1.0 (0.0)
*g*_known_ = 4				
UUU	4	UUC	1.0 (0.0)	NA
UUC	4	UUC	1.0 (0.0)	NA
UCU	4	UCC	1.0 (0.0)	NA
UCC	4	UCC	1.0 (0.0)	NA
CCU	5.1 (1.3)	CCU	0.958 (0.057)	1.0 (0.055)
CCC	5.1 (1.1)	CCU	0.960 (0.052)	1.0 (0.029)

Average ARI, most chosen model, and the average number of observation clusters selected for the remaining six OSGaBI models using simulated data with medium variance and good cluster separation when fitting *G*=2,…,10 observation clusters using 100 data sets and 20 random starts. The last column presents the ARI when fixing *G*=*g*_known_, where *g*_known_ represents the number of observation clusters the data was generated from, for the CCU and CCC models. Values in brackets represent the respective standard deviation

It is important to note that although the algorithm was overfitting for the number of components based on the model selected, the majority of the time the original components were simply being broken into smaller components. A classification table from one of the simulation results illustrates the very common occurrence (Table 4). In this specific result, Cluster 1 was broken up into three components by the algorithm, resulting in a total of four components. The final column of Tables 2 and 3 provide further evidence because once the algorithm is provided the correct number of components, the ARI become perfect or near perfect.

**Table 4:** An example of a classification table from one of the simulation results

	True			
	1	2	3	4
Estimated 1	56	32	12	0
2	0	0	0	100

Although the algorithm was overfitting for the number of components based on the model selected, the majority of the time the original components were simply being broken into smaller components

## Application

### Rat data

We present the biclustering results from Affymetrix oligonucleotide array data from a nutritional and pharmaceutical intervention in diabetic rats. This study consisted of five male lean control rats and five male Zucker diabetic fatty (ZDF) rats, which are genetically predisposed to developing diabetes. Details regarding the original rat study are described in Beaudoin et al. [35]. From each animal, tissue was extracted from various tissue depots, including the liver and red tibialis anterior (red TA, a type of muscle). Blood was also extracted, resulting in a total of 30 samples. RNA was extracted from these samples and used for the subsequent microarray gene expression analysis. Pre-processed data can be found on Gene Expression Omnibus (GEO) [36], accession number GSE93402 (blood), GSE93403 (liver), and GSE93406 (red TA). After pre-processing using the affy and oligo packages [37, 38] respectively for R Bioconductor [39, 40] respectively, *n*=8801 genes remained. We worked with the top 2000 differentially expressed genes between the red TA and liver (*p*<0.01). For this analysis, we set the genes as the observations (*n*=2000) and the samples as the variables (*p*=30).

The goal of the biclustering analysis was to identify sets of genes within the blood that possess similar expression profiles within the distinct tissues. Thus, we aimed to match biclusters containing genes that had similar expression profiles that were unique for blood and a specific tissue type. We focus here on genes with similar expression profiles between blood and liver. Downstream, these candidate genes can be tested to determine if they can function as blood biomarkers of metabolic status in individuals in different contexts (i.e., response to interventions, different disease states, etc.); however, this subsequent analysis goes beyond the scope of the present article.

We constrained the structure of ***Λ***_*g*_ because we knew the relationships required among the three sample types. Specifically, we wanted correlated expression between blood and liver only, implying that expression between blood and red TA were uncorrelated and expression between liver and red TA were uncorrelated as well. The other (extraneous) relationship characterized by the block-diagonal covariance matrix was the correlated nature of the expression strictly among the liver samples. Consequently, *q*=2 for the number of variable clusters (i.e., the two relationships described previously). Sample types were constant across all components, i.e., ***Λ***_*g*_=***Λ***, and thus we limited the algorithm to fit model CUU and CUC. These two *Λ*-constrained models were chosen based on the results from the simulation studies previously mentioned. We normalized the data and fitted the range of *G*=2,…,30.

**Fig. 2:**
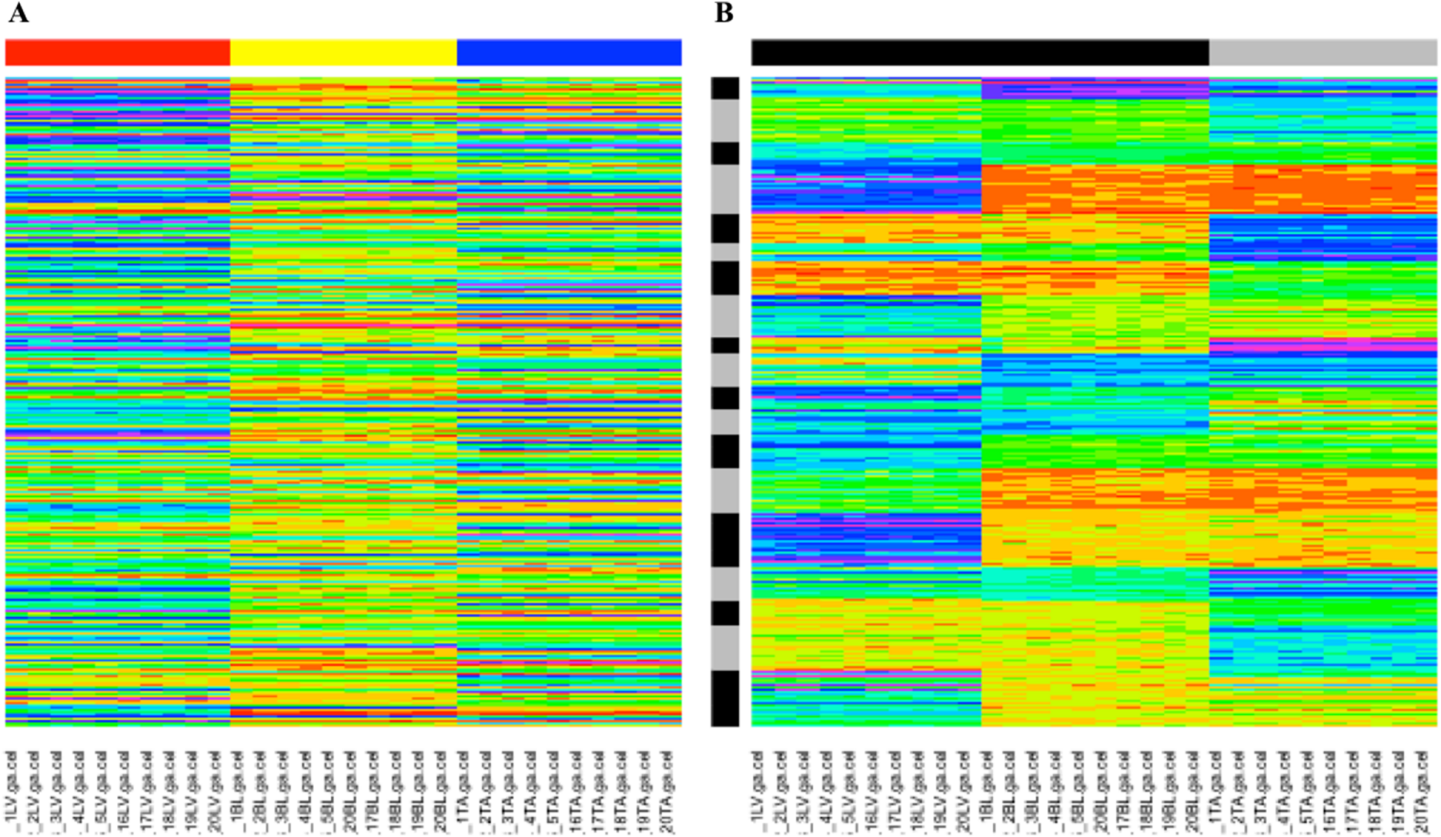
Heatmaps of the rat data. Heatmap of the rat data before biclustering (**a**). The *red*, *yellow*, and *blue bars* along the columns and represent liver, blood, and red TA samples, respectively. Heatmap of the rat data after biclustering and subsequently rearranging the rows so that the observation clusters were contiguous (**b**). *Black* and *grey bars* along the columns and rows simply represent the presence of the clusters and do not indicate relationships between them. *G*=19 for the observation clusters and along the columns, *q*_1_=*q*_2_=2

The BIC selected a CUU model with *G*=19 observation clusters for the blood-liver analysis. As seen from the heatmaps before and after biclustering and subsequent rearranging, there were definitive biclusters in the data (Fig. 2). We inputted the gene lists for each of the 19 biclusters into the online functional annotation tool DAVID (Database for Annotation, Visualization and Integrated Discovery) [41, 42] to elucidate potential biological processes that were dominant in each bicluster. DAVID functional annotation results indicated that the largest proportions of genes in the blood-liver biclusters had roles in protein metabolic and modification processes, carboxylic metabolic process, oxaloacid metabolic process, and intracellular signal transduction (biological processes as defined by the Gene Ontology Consortium, [43]), all biological processes of which have previously been shown to have an involvement in diabetes and obesity, and some processes within the liver [44–50]. These processes accounted for approximately 20–43% of the genes in the various biclusters and were all statistically significantly enriched (*p*<0.05). There is also a general inference that insulin resistance occurs at different times in insulin sensitive tissues such as muscle and liver [51, 52]; therefore, it is not surprising that the expression profiles between the liver and red TA were not similar. Additionally, it has been previously established that the peripheral blood transcriptome reflects changes in various tissues throughout the body [3], a property that is illustrated in the biclusters of interest.

### Human data

The second data set we analyzed is another Affymetrix oligonucleotide array retrieved from GEO, accession number GSE1133. The original study aimed to profile 79 human and 61 mouse tissues in terms of their transcriptomes under normal conditions [53]. Here, we focus on the human arrays, specifically the tissues related to the immune system (20 tissue types) and the brain (16 tissue types), and also whole blood, for a total of 37 tissue types. Each tissue had two replicates, giving a total of 74 samples. After pre-processing using the same methods described for the rat data and removing genes without Entrez gene identifiers, *n*=3867 genes remained, of which 2148 genes were differentially expressed between brain and immune tissues (*p*<0.01). Similar to the rat data, we set the genes as the observations (*n*=2148) and the samples as the variables (*p*=74).

The goal of this biclustering analysis was to identify sets of genes within the blood that possess similar expression within the distinct groups of tissues. Thus, we aimed to match biclusters containing genes that had similar expression that were unique for blood and a specific group of tissues. We focus here on genes with similar expression between blood and immune tissues. Subsequent work can involve determining which of these candidate genes can function as blood biomarkers of normal immune function in individuals.

Similar to the rat data, we constrained the structure of ***Λ***_*g*_ because we knew the relationships required among the three sample types. Specifically, we wanted correlated expression only between blood and immune tissues. This implied that expression between blood and brain tissues were uncorrelated, and expression between immune and brain tissues were uncorrelated as well. The other (extraneous) relationship characterized by the block-diagonal covariance matrix was the correlated nature of the expression strictly among the immune tissue samples. Consequently, *q*=2 for the number of variable clusters (i.e., the two relationships described previously). Samples were constant across all components, i.e., ***Λ***_*g*_=***Λ***, and thus we again limited the algorithm to fit model CUU and CUC. We normalized the data and fitted models in the range of *G*=2,…,30.

**Fig. 3:**
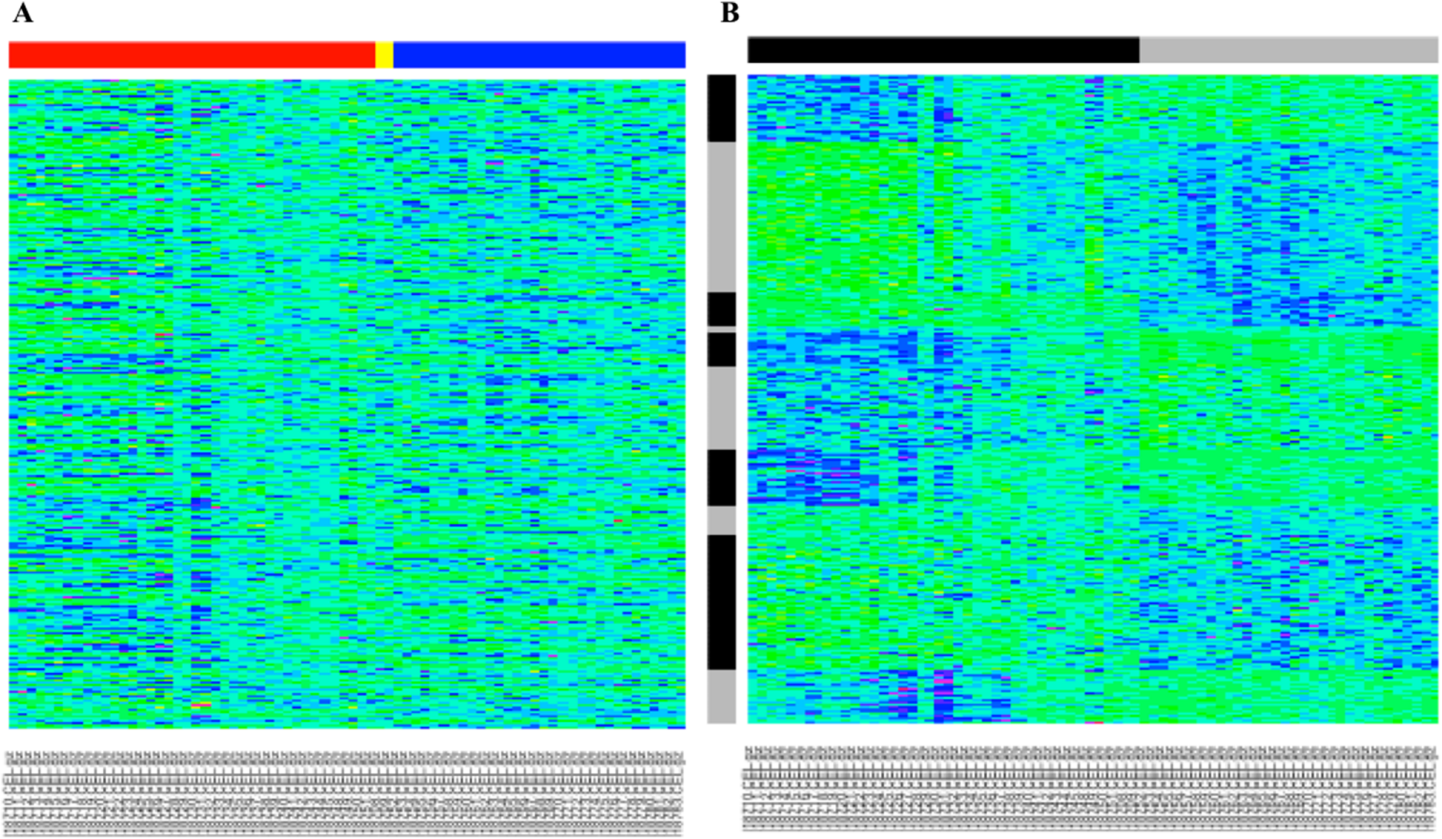
Heatmaps of the human data. Heatmap of the human data before biclustering (**a**). The *red*, *yellow*, and *blue bars* along the columns and represent immune tissues, whole blood, and brain tissues respectively. Heatmap of the human data after biclustering and subsequently rearranging the rows so that the observation clusters were contiguous (**b**). *Black* and *grey bars* along the columns and rows simply represent the presence of the clusters and do not indicate relationships between them. *G*=10 for the observation clusters and along the columns, *q*_1_=*q*_2_=2

The BIC selected a CUU model with *G*=10 observation clusters for the blood-immune analysis. As seen from the heatmaps before and after biclustering and subsequent rearranging, there were again definitive biclusters in the data (Fig. 3). DAVID functional annotation results indicated that the largest portion of genes in each bicluster had roles in the nucleobase-containing small molecule metabolic process, macromolecule metabolic process, microtubule-based process, microtubule cytoskeletal organization, response to DNA damage stimulus, and transmembrane transport; all biological processes that have been linked to immune responses [54–57]. These processes accounted for anywhere between 4 to 51% of the genes in the various biclusters, and were all statistically significantly enriched (*p*<0.05). Furthermore, blood acts as a transporter for the immune system by transporting immune cells throughout the body, thus blood can provide an extensive view of the immune status of an individual [58]. This property is reflected in the biclusters of interest because there is a correlation among the expression between the blood and the immune tissues.

## Discussion

One specific taxonomy of biclustering methods for gene expression data aims to retrieve non-overlapping biclusters characterized by one specific sample type (in this case, “sample” could refer to a type of treatment, tissue, disease state, etc.) along the variable dimension, as reviewed in Pontes et al. [19]. This is useful in applications such as disease subtype discovery, where the focus is to elucidate the various disease subtypes based on the genes. Conversely, in blood biomarker discovery, knowledge of the types of samples a priori is required and the focus is on the relationships between those sample types based on the genes, which is where one-way supervised biclustering is able to play a role. Two inherent criteria of blood biomarkers are that there is 1) a correlation between blood and the tissue of interest and 2) no correlation between those two sample types and other tissues. The second criteria is enforced by including other tissues into the biclustering analysis so that the condition can be set in conjunction with the first criteria. The relationships required are satisfied through the use of one-way supervision to explicitly determine the relationship between blood and the various tissues. To the best of our knowledge, biclustering methods currently available under the taxonomy of non-overlapping biclusters do not provide the option of one-way supervision along the variable dimension to aid in applications such as blood biomarker discovery.

Another advantage of approaching tissue-specific blood biomarker discovery through the use of biclustering is the ability to identify groups of genes that are potentially related to each other through their biological pathways. Commonly, correlation analysis between blood and a tissue is conducted using the available gene list in its entirety, e.g. [59], consequently not revealing any information about genes related by biological pathways that a cluster analysis would provide. In our OSGaBi family, setting the variable clusters labels and subsequently biclustering conditional on this information allows us to handle this limitation of correlation analysis.

Simulation study results show that models with values of *G* that are too high are sometimes selected, and this problem becomes more pronounced for high variance. While the BIC has been shown to be unreliable in higher dimensions, e.g. [60] — and this may suggest that further research on an optimal model selection criteria for this family of biclustering models is warranted — it is quite possible that the selection of larger values of *G* is simply a result of lack of concentration around the modes at higher variances. The inclusion of results for fixed *G* follows [61] and [62], who carried out mixture model analysis of gene expression data by treating *G* as fixed and known. Note that, in [23] where the binary row-stochastic factor loadings matrix is a property of their MFABC family, the authors report simulation results but do not mention the model selection criterion or the range of number of observation clusters fitted; therefore, it is not known if the authors treated *G* as fixed. Conversely, the authors mention the use of the BIC and AIC for model selection in their real data study with gene expression data, supporting the use of the BIC for our analyses until the optimal model selection criteria is determined.

Future work will also aim to compare performance of the OSGaBi family to that of other model-based biclustering algorithms capable of detecting non-overlapping clusters and allowing for one-way supervision. Current methods are available for the former (as mentioned previously), but do not allow for the latter criteria. This limitation in the existing methods makes it difficult to compare the genes that are found in the biclusters to those found using the OSGaBi family since they do not always correspond to the intended subset of variables.

We have presented biclustering results using the OSGaBi family on two real microarray gene expression data sets. The first one was a previously unpublished rat microarray gene expression data set, where identified biclusters corresponded to genes whose expression profiles were correlated between liver and blood (and not between red TA and blood, or liver and red TA). Identified biclusters were enriched in genes related to biological processes known to play a role in insulin resistance and obesity in a tissue-specific manner. The second data set was a subset of a microarray gene expression data set from the GEO database that aimed to profile the human transcriptome under normal conditions. In this analysis, identified biclusters corresponded to genes whose expression correlated between immune tissues and blood (and not between brain tissues and blood, or immune and brain tissues). Identified biclusters contained genes related to biological processes previously associated with the immune system. Although further biological experimental analysis and interpretation need to be conducted to determine the best candidate gene(s) in both preliminary analyses, the initial results show promise in using the OSGaBi biclustering family for discovering novel blood biomarkers to act as surrogate tissue material in the maintenance of health and the prevention of disease.

## Conclusions

A family of parsimonious Gaussian mixture models for the biclustering of gene expression data has been proposed. These models work in a one-way-supervised fashion in that the variable labels are known. The binary and row-stochastic factor loadings matrix results in a block-diagonal covariance matrix, which can be a useful property in biclustering applications for dictating the relationships between the variables. A promising application for our method is in the discovery of novel peripheral blood biomarkers for use as surrogate biopsy material.

## Additional file

Additional file 1 Supplementary files. (PDF 90 kb)

## Abbreviations

AECM: Alternating expectation-conditional maximization
AIC: Akaike information criterion
ARI: Adjusted rand index
BIC: Bayesian information criterion
DAVID: Database for annotation: visualization and integrated discovery
EM: Expectation-maximization
GEO: Gene expression omnibus
ICL: Integrated completed likelihood
MAP: Maximum *a posteriori*
MFA: Mixtures of factor analyzers
MFABC: Mixtures of factor analyzers for biclustering
MLE: Maximum likelihood estimates
OSGaBi: One-way supervised Gaussian biclustering
PGMM: Parsimonious Gaussian mixture model
PPCA: Probabilistic principal components analysis
TA: Tibialis anterior
ZDF: Zucker diabetic fatty
