# Supplementary material for: Two-way learning with one-way supervision for gene expression data
Source: BMC Bioinformatics. 2017 Mar 4;18:150. doi: 10.1186/s12859-017-1564-5 (PMC5336648; doi:10.1186/s12859-017-1564-5)
Supplement: Additional file 1 — Supplementary files. (PDF 90 kb) [file 12859_2017_1564_MOESM1_ESM.pdf]

Appendix

The resulting AECM parameter updates for the remaining OSGaBi models are listed below.

Model UUU

$\boldsymbol{\beta}_g = \boldsymbol{\Lambda}_g'(\boldsymbol{\Lambda}_g\boldsymbol{\Lambda}_g' + \boldsymbol{\Psi}_g)^{-1}$
$\boldsymbol{\Theta}_g = \mathbf{I}_q - \boldsymbol{\beta}_g\boldsymbol{\Lambda}_g + \boldsymbol{\beta}_g\mathbf{S}_g\boldsymbol{\beta}_g'$
$\hat{\boldsymbol{\Psi}}_g = \text{diag}\left\{\mathbf{S}_g - \boldsymbol{\Lambda}_g\boldsymbol{\beta}_g\mathbf{S}_g\right\}$

Model UCU

$\boldsymbol{\Psi}_g = \boldsymbol{\Psi}$

$$\boldsymbol{\beta}_g = \boldsymbol{\Lambda}_g'(\boldsymbol{\Lambda}_g\boldsymbol{\Lambda}_g' + \boldsymbol{\Psi})^{-1}$$
$$\mathbf{S}_g = \frac{1}{n_g}\sum_{i=1}^{n}\hat{z}_{ig}(\mathbf{x}_i - \boldsymbol{\mu}_g)(\mathbf{x}_i - \boldsymbol{\mu}_g)'$$
$$\boldsymbol{\Theta}_g = \mathbf{I}_q - \boldsymbol{\beta}_g\boldsymbol{\Lambda}_g + \boldsymbol{\beta}_g\mathbf{S}_g\boldsymbol{\beta}_g'$$
$$\hat{\boldsymbol{\Psi}} = \sum_{g=1}^{G}\hat{\pi}_g \ \text{diag}\left\{\mathbf{S}_g - \boldsymbol{\Lambda}_g\boldsymbol{\beta}_g\mathbf{S}_g\right\}$$

Model UUC

$\boldsymbol{\Psi}_g = \psi_g\mathbf{I}_p$

$$\boldsymbol{\beta}_g = \boldsymbol{\Lambda}_g'(\boldsymbol{\Lambda}_g\boldsymbol{\Lambda}_g' + \psi_g\mathbf{I}_p)^{-1}$$
$$\hat{\psi}_g = \frac{1}{p}\text{tr}\left\{\mathbf{S}_g - \boldsymbol{\Lambda}_g\boldsymbol{\beta}_g\mathbf{S}_g\right\}$$

Model UCC

$\boldsymbol{\Psi} = \psi\mathbf{I}_p$

$$\boldsymbol{\beta}_g = \boldsymbol{\Lambda}_g'(\boldsymbol{\Lambda}_g\boldsymbol{\Lambda}_g' + \psi\mathbf{I}_p)^{-1}$$
$$\hat{\psi} = \frac{1}{p}\sum_{g=1}^{G}\hat{\pi}_g\text{tr}\left\{\mathbf{S}_g - \boldsymbol{\Lambda}_g\boldsymbol{\beta}_g\mathbf{S}_g\right\}$$

Model CCU

$\boldsymbol{\Lambda}_g = \boldsymbol{\Lambda}$, $\boldsymbol{\Psi}_g = \boldsymbol{\Psi}$

$$\boldsymbol{\beta} = \boldsymbol{\Lambda}'(\boldsymbol{\Lambda}\boldsymbol{\Lambda}' + \boldsymbol{\Psi})^{-1}$$
$$\mathbf{S} = \sum_{g=1}^{G}\hat{\pi}_g\mathbf{S}_g$$
$$\boldsymbol{\Theta} = \mathbf{I}_q - \boldsymbol{\beta}\boldsymbol{\Lambda} + \boldsymbol{\beta}\mathbf{S}\boldsymbol{\beta}'$$
$$\hat{\boldsymbol{\Psi}} = \text{diag}\left\{\mathbf{S} - \boldsymbol{\Lambda}\boldsymbol{\beta}\mathbf{S}\right\}$$

Model CUC

$$
\begin{aligned}
&\boldsymbol{\Lambda}_g = \boldsymbol{\Lambda},\ \boldsymbol{\Psi}_g = \psi_g \mathbf{I}_p \\
&\quad \boldsymbol{\beta}_g = \boldsymbol{\Lambda}'(\boldsymbol{\Lambda}\boldsymbol{\Lambda}' + \psi_g \mathbf{I}_p)^{-1} \\
&\quad \hat{\psi}_g = \frac{1}{p}\mathrm{tr}\left\{\mathbf{S}_g - 2\boldsymbol{\Lambda}\boldsymbol{\beta}_g\mathbf{S}_g + \boldsymbol{\Lambda}\boldsymbol{\Theta}_g\boldsymbol{\Lambda}'\right\}
\end{aligned}
$$

Model CCC

$$
\begin{aligned}
&\boldsymbol{\Lambda}_g = \boldsymbol{\Lambda},\ \boldsymbol{\Psi} = \psi \mathbf{I}_p \\
&\quad \boldsymbol{\beta} = \boldsymbol{\Lambda}'(\boldsymbol{\Lambda}\boldsymbol{\Lambda}' + \psi \mathbf{I}_p)^{-1} \\
&\quad \hat{\psi} = \frac{1}{p}\mathrm{tr}\left\{\mathbf{S} - \boldsymbol{\Lambda}\boldsymbol{\beta}_g\mathbf{S}\right\}
\end{aligned}
$$
